# Expanding the Catalog of Patient and Caregiver Out-of-Pocket Costs: A Systematic Literature Review

**DOI:** 10.1089/pop.2023.0238

**Published:** 2024-02-06

**Authors:** Theresa Schmidt, Christine Juday, Palak Patel, Taruja Karmarkar, Esther Renee Smith-Howell, A. Mark Fendrick

**Affiliations:** ^1^ Real Chemistry, New York, New York, USA.; ^2^ Merck & Co., Inc., Rahway, New Jersey, USA.; ^3^ University of Michigan School of Medicine/School of Public Health, Ann Arbor, Michigan, USA.

**Keywords:** out-of-pocket, patient costs, caregiver burden, cost sharing, indirect costs, direct costs

## Abstract

Out-of-pocket (OOP) health care expenditures in the United States have increased significantly in the past 5 decades. Most research on OOP costs focuses on expenditures related to insurance and cost-sharing payments or on costs related to specific conditions or settings, and does not capture the full picture of the financial burden on patients and unpaid caregivers. The aim for this systematic literature review was to identify and categorize the multitude of OOP costs to patients and unpaid caregivers, aid in the development of a more comprehensive catalog of OOP costs, and highlight potential gaps in the literature. The authors found that OOP costs are multifarious and underestimated. Across 817 included articles, the authors identified 31 subcategories of OOP costs related to direct medical (eg, insurance premiums), direct nonmedical (eg, transportation), and indirect spending (eg, absenteeism). In addition, 42% of articles studied an expenditure that the authors did not label as “OOP.” A holistic and comprehensive catalog of OOP costs can inform future research, interventions, and policies related to financial barriers to health care in the United States to ensure the full range of costs for patients and unpaid caregivers are acknowledged and addressed.

## Introduction

Total out-of-pocket (OOP) health care expenditure has grown significantly in the United States, increasing from $24 billion in 1970^*^ to $433 billion in 2021.^1,2^ Survey results from 2023 indicate that 86% of adults across the United States agree that decreasing OOP costs should be a national priority.^3^ OOP costs result in financial burden to patients and their families, with about half of American adults experiencing challenges in affording routine health care costs such as copayments for prescription medications.^4,5^

A Centers for Disease Control and Prevention (CDC) study reported 8.2% of adults aged 18–64 years not taking a prescription medication as prescribed due to cost. The proportion was even higher among individuals with a disability (20.0%), fair or poor health (18.0%), or without insurance (22.9%).^6^ Limited availability of low-cost premiums for public health insurance coverage also present a barrier to obtaining and maintaining insurance coverage for low-income individuals.^7^

Although OOP costs represent a national issue, they do not impact all patients equally. High costs for health care services disproportionately impact adults without insurance, low-income populations, and Hispanic and Black adults in the United States.^4^ The impacts of systemic racism and structural inequities have produced higher levels of poverty among Black individuals, Hispanic/Latina/Latino/Latinx individuals, and other historically excluded populations.

Such racism and inequities have further created unequal exposures to social determinants of health (SDOH) that contribute to poor health and a higher prevalence of chronic conditions among marginalized individuals.^8^ A larger proportion of Black and Hispanic as well as low-income adults have postponed or forgone health care due to cost compared with White and higher income individuals.^4^ Analyzing OOP costs in relation to SDOH provides a more nuanced understanding for how these costs differentially impact certain individuals.

Public and private policies can impact OOP costs and, thus, either exacerbate disparities and inequities or promote more equitable care and outcomes. Whether through a court decision with the potential to strike down the preventive services provisions of the Affordable Care Act,^9^ lawmakers passing statutes, government agencies creating payment programs, employers providing employees health insurance, payers making coverage and cost-sharing decisions, pharmacy benefit managers implementing accumulators, or life sciences companies implementing patient access programs, the impact of policies on the type and volume of OOP costs assumed by different patients and families cannot be overstated.

Levels of cost sharing (eg, high deductibles, copays, coinsurance) have increased for the past few decades as insurers have attempted to reduce unnecessary utilization; these changes have been associated with increases in medical debt.^10^ Higher OOP costs, including insurance and cost-sharing payments, are related to patient decision making and health behaviors such as decreased medication adherence, lower utilization of preventive care services, and delayed or missed medical care.^11–13^

Research shows that almost 40% of Americans reported postponing medical treatment due to cost in 2022; this represents the highest percentage for the past 2 decades and compares with 26% in 2020.^14^ Even relatively small levels of cost sharing are related to decreased use of necessary care.^15^ OOP costs other than direct medical and insurance costs also impact patient care decisions and behaviors. For example, transportation costs are significant barriers to health care access, especially among individuals with lower socioeconomic status.^16,17^

Research to date has mostly limited the measurement of OOP expenditures to insurance and direct payments for care.^18,19^ Researchers also often employ a disease- or outcome-specific lens when studying OOP costs. For example, several studies have been published investigating OOP costs in cancer care and the impact of OOP costs on specific outcomes such as medication adherence or utilization of preventive services.^12,20–22^

Moreover, literature on the relationship between OOP costs and patient characteristics is limited to a few variables, including socioeconomic status, age, and insurance type. Similarly, previous literature on OOP costs to unpaid caregivers (eg, family members) largely focuses on specific costs, clinical areas, and/or populations.^23–28^

Current definitions of health care OOP costs, mainly focused on insurance, do not provide the full picture of health-related OOP burden on patients and unpaid caregivers, and there is no systematic literature review that comprehensively catalogs OOP costs across therapeutic areas and populations for patients and unpaid caregivers.

Although previous research is important for explaining financial barriers to specific types of care and can inform necessary policies that address the high cost of insurance and cost-sharing payments, a more comprehensive real-world understanding of OOP expenditures (ie, costs experienced by actual patients and/or unpaid caregivers related to health care) can lead to more accurate estimates of the magnitude of and tolerance for financial burden.

Accordingly, the authors performed a systematic literature review to identify the multitude of OOP costs for patients and unpaid caregivers captured in existing literature and potential gaps for future research. The authors intended to inventory OOP costs to better demonstrate the universe of OOP burdens faced by patients and unpaid caregivers in the United States. By establishing a more comprehensive catalog of OOP costs, future qualitative and quantitative research could be conducted to illuminate patient perspectives and encourage a more informed approach toward patient OOPs by policymakers, along with public and private entities delivering health care.

## Materials and Methods

The authors used the Preferred Reporting Items for Systematic Reviews and Meta-Analyses (PRISMA) guidance and the PRISMA 2020 Item Checklist to produce a transparent, complete, and accurate account of why the systematic review was conducted, how the study was conducted, and the findings.^29^

### Search strategy and selection criteria

The authors performed a systematic search of PubMed, Cochrane Central Register of Controlled Trials, and Embase using Boolean logic to identify relevant articles. See Supplementary Appendix SA1 for the search strings. The conceptual model used to inventory patient characteristics and OOP costs was based on the Sample, Phenomenon of Interest, Design, Evaluation, Research type framework, with the addition of other limiters, as noted in Supplementary Appendix SA2.^30^

The current review included English-language articles published between April 1, 2017 and March 31, 2022, which defined direct and/or indirect OOP costs associated with real-world health care experiences specific to actual US patients and/or unpaid caregivers. The authors defined real-world health care experiences as those reported by patients and unpaid caregivers in real-life settings or based on other patient-level data (eg, claims). Experiences reported by other stakeholders (eg, physicians, nurses) were included if the article explicitly noted that patients and/or their unpaid caregivers discussed these experiences with the stakeholders.

The authors excluded conference abstracts, literature and narrative reviews with no original research, study protocols, clinical trials, commentaries/opinion articles, and cost-effectiveness studies. The authors also excluded articles examining theoretical frameworks for medication pricing or assessing medication pricing variation, hypothetical results, cost estimates from economic analyses, and policy analysis predicting future impacts.

Covidence, a not-for-profit service working in partnership with Cochrane to improve the development and use of systematic review for health care, was used to store and screen articles.^31^ Reviewers applied inclusion and exclusion criteria to titles, abstracts, and full texts, with at least 2 reviewers independently screening each article.

### Data extraction

Reviewers independently extracted study/population characteristics and OOP costs from included articles using a collaborative web-based data collection tool built for this purpose with Microsoft 365 Excel exporting capabilities. Study/population characteristics data included basic article information (eg, title, authors), data source, data collection period, author's objective (including whether it includes studying OOP), sociodemographic characteristics of the study population (eg, US region, age), and clinical characteristics (eg, primary therapeutic area).

OOP cost data included the type of OOP cost, whether the OOP cost was to patients and/or unpaid caregivers, whether the author specifically referred to it as an “out-of-pocket” cost, and if the cost was stratified by sociodemographic, clinical, and/or economic characteristics. An article could have more than 1 OOP cost, each of which was independently extracted.

The authors performed a quality assurance review on each extraction to ensure accuracy of extracted information. Disagreements during screening and extraction were resolved by an adjudicator, distinct from the article reviewers, and, if needed, by achieving consensus among all parties.

### Data synthesis and analysis

After extraction, the authors performed thematic analysis to identify key patterns across included articles. This included classifying study/population characteristics and OOP costs into subcategories developed *a priori* and refined based on extracted information. Microsoft Excel 365 was used to calculate descriptive statistics of key variables, such as data source and study type.

Articles were categorized based on key study and population characteristics. Categorizations for geography, data source, and sociodemographic and clinical characteristics were not mutually exclusive. This led to results showing a sum in each category that is more than the number of included articles. Other data, including study type and article objective, were mutually exclusive.

Although acknowledging that the categorization of costs is inconsistent across literature, the authors identified 3 main categories of OOP costs for this review: (1) “direct medical costs” included OOP costs for medical care/treatment charged directly to patients and/or unpaid caregivers (eg, insurance copay for a doctor's visit); (2) “direct nonmedical costs” included additional OOP costs obtained while accessing or receiving care/treatment (eg, expenses related to travel to appointments); and (3) “indirect costs” included any earnings or savings lost by patients or unpaid caregivers due to health or while seeking care/treatment (eg, absenteeism).^32–35^ Studies for which reported OOP costs could not be differentiated into categories were not included in the cost categorization and related analysis.

The authors did not conduct a meta-analysis due to heterogeneity across the volume of literature analyzed including diversity in study design, geography, populations, and OOP costs.

## Results

**FIG. 1.**
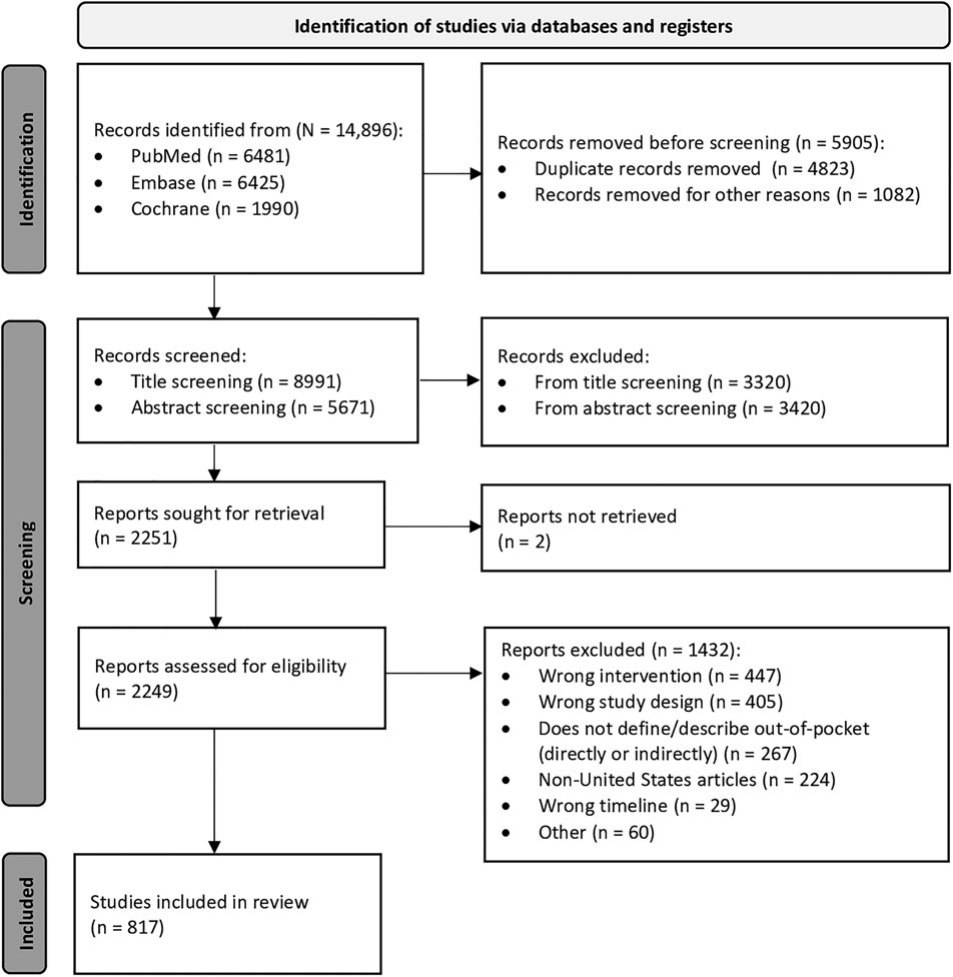
PRISMA flow diagram. A systematic search of PubMed, Cochrane Central Register of Controlled Trials, and Embase identified 14,896 total articles. After abstract and full-text review, 817 articles met the inclusion criteria and were included in the review for further analysis.

The initial search identified 14,896 articles. After abstract and full-text review, 817 articles met the inclusion criteria and were included for analysis (Fig. 1).

### Article study type

Of the 817 articles included in the review, the most common study type was prospective and/or retrospective cohort (*n* = 272), followed by cross-sectional (*n* = 167) and studies using only qualitative methodologies (*n* = 73). The most common data sources were surveys (*n* = 337), claims/billing data (*n* = 305), and focus groups (*n* = 107). Two articles featured qualitative analysis of social media data. For the 423 articles that used surveys or a qualitative data source (eg, focus groups, interviews), patients were the most common respondents (*n* = 355), followed by family/unpaid caregivers (*n* = 100) and clinicians (*n* = 50). As noted earlier, article counts for data sources were not mutually exclusive.

### Article objective

Of the 817 articles, 83% (*n* = 676) studied OOP costs as part of their principal objective. For the remaining 17% (*n* = 141), OOP costs were captured through inquiry focused on other areas or as secondary variables. Some of these articles examined health care utilization or barriers to care, whereas others evaluated specific care programs. An example of a non-OOP study objective includes the following: “To examine the prevalence and health care use of acute respiratory tract infections among cancer survivors in the United States in a population-based cohort.”^36^

### Population characteristics

Many articles limited their study populations based on sociodemographic characteristics. For example, 94 articles specified populations limited to only English speakers, 22 limited to English and Spanish speakers, and 3 included populations speaking other languages (ie, French, Hmong, Vietnamese). Articles were also more likely to study adults (ie, specified including individuals 18 years or older) (*n* = 550) compared with pediatric populations (ie, specified including individuals younger than 18 years) (*n* = 88).

Only 11 articles (1%) limited their study populations by race or ethnicity, with 3 limited to Black or African American and White individuals; 2 limited to Hispanic/Latino; 2 to Black/African American; 1 Black/African American and Hispanic/Latino; 1 to Asian; 1 to Asian and White; and 1 to American Indian/Alaskan Native individuals. Eighty-eight articles (11%) limited study populations to women only, 5 men only, and 2 transgender/nonbinary individuals only. In addition, 157 articles limited their study population to individuals with commercial insurance only, 13 to Medicaid only, 62 to Medicare only, and 14 to those without insurance.

Lastly, 428 articles studied populations across all regions and 261 articles studied populations across all 50 states. Among articles that specified a region (but not all regions, *n* = 214), the largest proportion studied populations in the South (33%) and the smallest studied populations in the Midwest (22%). Among 220 articles that specified a state (but not all states), the largest proportion studied populations in California (20%) or Massachusetts (14%). As noted earlier, article counts for geography as well as other sociodemographic and clinical characteristics were not mutually exclusive. See Supplementary Appendix SA3 for more details on article characteristics.

### Categorizing OOP costs

Of 817 articles, 71% referred to at least 1 identified cost specifically as “out-of-pocket,” and 42% included at least 1 cost that the current review defined as OOP, but the article did not refer to as “out-of-pocket.”

In addition, 808 (99%) had costs that could be further grouped into direct medical, direct nonmedical, and indirect costs. The remaining 9 articles captured “all OOP” but did not list specific costs. See Table 1 for a complete catalog of the categorized OOP costs. Direct medical costs are studied in 656 (81%) articles, direct nonmedical costs in 223 (28%) articles, and indirect costs in 206 (25%) articles.

**Table 1.** Categorization and Subcategorization of the Different Types of Out-of-Pocket Costs (Listed Alphabetically)^a^

OOP cost name (n, % of articles)	Cost description	No. of articles referring to cost as “OOP” (%)	No. of articles studying costs to patients (%)	No. of articles studying costs to unpaid caregiver (%)
Direct medical costs				
Adaptive/medical equipment costs (*n* = 25, 3%)	OOP costs for medical devices or equipment (durable and other). Examples include costs for medical equipment, vision aids, prostheses, and mobility devices.	20 (80%)	23 (92%)	13 (52%)
Dental care costs (*n* = 27, 3%)	OOP costs related to dental care or dental services	24 (89%)	26 (96%)	11 (41%)
Diagnosis/treatment/management costs (beyond cost sharing) (*n* = 197, 24%)	OOP costs for allopathic health care services that are not explicitly defined as cost sharing (copayment, co-insurance, deductible), excluding medications and dental care. Examples include costs related to lab work, diagnostic testing, consultation, rehabilitation, preventative care/screening, autism spectrum disorder-specific agency services, medical supplies, and self-pay for weight management program.	136 (69%)	179 (91%)	58 (29%)
Home health/home care costs (*n* = 20, 2%)	OOP costs for home health and home care	20 (100%)	19 (95%)	10 (50%)
Hospice care costs (*n* = 3, 0.4%)	OOP costs for hospice care services	3 (100%)	3 (100%)	1 (33%)
Insurance premiums (*n* = 34, 4%)	OOP costs for insurance premiums	21 (62%)	32 (94%)	16 (47%)
Insurance cost sharing for medication (*n* = 281, 34%)	Payments explicitly defined as cost sharing (copayment, co-insurance, deductible) for medications	248 (88%)	274 (98%)	26 (9%)
Insurance cost sharing for nonmedication (*n* = 280, 34%)	Payments explicitly defined as cost sharing (copayment, co-insurance, deductible) for health care, excluding medications and dental care	235 (84%)	266 (95%)	43 (15%)
Long-term care facility costs (*n* = 10, 1%)	OOP costs for long-term care facilities	10 (100%)	10 (100%)	5 (50%)
Medical supply costs (*n* = 9, 1%)	OOP costs for disposable and/or routine medical supplies (eg, test strips, bandages, compression garments, insulin pumps)	7 (78%)	8 (89%)	5 (56%)
Medication costs (beyond cost sharing) (*n* = 168, 21%)	OOP costs for medications and prescription medications that are not explicitly defined as cost sharing (copayment, co-insurance, deductible)	118 (70%)	161 (96%)	40 (24%)
Over-the-counter product costs (*n* = 23, 3%)	OOP costs for over-the-counter medications and other health-related products	15 (65%)	19 (83%)	9 (39%)
Professional caregiver costs (*n* = 5, 0.6%)	OOP costs for professional caregivers and nurses	2 (40%)	5 (100%)	0 (0%)
Direct nonmedical costs				
Accommodation costs (*n* = 30, 4%)	OOP costs for housing or accommodations for health care services/treatments	18 (60%)	24 (80%)	11 (37%)
Alternative and complementary therapy costs (*n* = 16, 2%)	OOP costs for treatment or care that do not fall under traditional allopathic practices and any cost specifically defined as alternative or complementary by article. Examples include costs for homeopathy, massage, Ayurveda, acupuncture, yoga, chiropractic services, and home remedies.	11 (69%)	12 (75%)	8 (50%)
Child/dependent care costs (*n* = 55, 7%)	OOP costs for childcare, elder care, or care for any other dependent(s)	28 (51%)	39 (71%)	32 (58%)
Clothing costs (*n* = 6, 0.7%)	OOP costs related to clothing bought due to health condition or health management. Example includes clothing purchased due to weight loss from a health condition.	4 (67%)	4 (67%)	4 (67%)
Education/academic support costs (*n* = 3, 0.4%)	OOP costs for education support needed due to a health condition	1 (33%)	1 (33%)	3 (100%)
Exercise and sports-related costs (*n* = 5, 0.6%)	OOP costs for sport-related services and items such as gym memberships	4 (80%)	5 (100%)	2 (40%)
Family/unpaid caregiver costs (*n* = 24, 3%)	OOP costs that were defined by articles as family or unpaid caregiver expenses	5 (21%)	10 (42%)	18 (75%)
Funeral costs (*n* = 1, 0.1%)	OOP costs for funeral expenses	1 (100%)	0 (0%)	1 (100%)
Household care costs and/or bills (*n* = 34, 4%)	OOP costs for household care services or bills (eg, utilities, Wi-Fi bills, home repairs)	17 (50%)	26 (76%)	22 (65%)
Legal costs (*n* = 5, 0.6%)	OOP costs for legal expenses related to receiving care or due to health condition	2 (40%)	3 (60%)	4 (80%)
Meal costs (*n* = 25, 3%)	OOP costs for meals bought due to patient receiving health care services or due to special diet or restrictions related to health condition	16 (64%)	15 (60%)	14 (56%)
Parking-related costs (*n* = 26, 3%)	OOP costs for parking for health care encounters	13 (50%)	20 (77%)	12 (46%)
Personal care costs (*n* = 6, 0.7%)	OOP costs for cosmetic or personal care related to a health condition. Examples include costs wigs/headwear, hair appointments, lotions, and other self-care	4 (67%)	6 (100%)	4 (67%)
Technology-related costs (*n* = 1, 0.1%)	OOP costs for electronic devices for telehealth visits	1 (100%)	1 (100%)	1 (100%)
Transportation/travel costs (*n* = 196, 24%)	Travel burden, including but not limited to costs for transportation, as defined by the article (eg, gas, bills related to ground or air transportation)	59 (30%)	167 (85%)	68 (35%)
Indirect costs				
Absenteeism/lost wages (*n* = 199, 24%)	Absences from work and/or lost wages due to absences	27 (14%)	154 (77%)	74 (37%)
Presenteeism/productivity (*n* = 68, 8%)	Lost productivity although present at work	5 (7%)	54 (79%)	23 (34%)
Retirement savings (*n* = 3, 0.4%)	Using retirement savings to pay for OOP costs, leading to lost income during retirement.	1 (33%)	2 (67%)	2 (67%)

OOP costs were categorized into direct medical costs, direct nonmedical costs, and indirect costs. Subcategories are listed alphabetically.

^a^ Some articles studied multiple OOP costs across multiple categories.

OOP, out-of-pocket.

The most frequently studied OOP cost types were insurance cost sharing for medication (*n* = 281) and insurance cost sharing for nonmedication (*n* = 280). Insurance cost sharing for medication was referred to as “out-of-pocket” in 88% of those articles and insurance cost sharing for nonmedication was referred to as “out-of-pocket” in 84% of those articles.

Although absenteeism/lost wages was the third most studied type of OOP cost (*n* = 199), only 14% of these articles referred to absenteeism/lost wages as “out-of-pocket.” Similarly, transportation/travel costs were studied in 196 articles, and only 30% of these articles referred to transportation/travel costs as “out-of-pocket.” Technology-related costs (*n* = 1) and exercise and sports-related costs (*n* = 5) represented some of the less commonly studied direct nonmedical costs.

In addition to the categories listed in Table 1, 20 articles mentioned that certain OOP costs were incurred by patients and unpaid caregivers either because the cost was not covered by insurance or because the service was delivered by an out-of-network provider.

### Measuring OOP costs

Of the 817 included articles, 93% discussed at least 1 OOP cost for patients (*n* = 762) and 23% discussed at least 1 OOP cost for unpaid caregivers (*n* = 184). For the 762 articles discussing costs to patients, insurance cost sharing for medication was the most frequently studied OOP cost (*n* = 274), followed by insurance cost sharing for nonmedication (*n* = 266).

Articles addressing costs to patients were also more likely to study a direct medical cost (82%), compared with a direct nonmedical (24%) or an indirect cost (21%). For the 184 articles discussing costs to unpaid caregivers, absenteeism/lost wages (*n* = 74) and transportation/travel (*n* = 68) were the most frequently studied OOP costs. These articles were also more likely to study direct nonmedical costs (56%) or indirect costs (49%), compared with direct medical costs (43%).

### OOP costs studied within specific clinical areas

Of the 817 total articles, 645 specified 1 or more clinical area that was studied, whereas 172 either did not specify a clinical area or were broadly applicable. Oncology was the most commonly studied clinical area (*n* = 177) and has articles with the most variation in types of OOP costs studied, with 29 different subcategories of OOP costs identified. Within these 177 articles, the most commonly studied costs were insurance cost sharing for nonmedication (41%), insurance cost sharing for medication (40%), and transportation/travel costs (27%).

Other clinical areas studied included mental and behavioral health (*n* = 65), neurology (*n* = 60), women's health/OB-GYN (*n* = 49), metabolism/endocrinology (*n* = 45), cardiology (*n* = 44), and infectious disease/vaccines/immunology (*n* = 44). Insurance cost sharing for medication was the most commonly studied OOP cost across each of these clinical areas, except women's health/OB-GYN where 41% studied insurance cost sharing for nonmedication. Orthodontics was one of the least commonly studied clinical areas and had the least variation in the types of costs studied. Only 1 article focused on orthodontics, studying diagnosis/treatment/management costs (beyond cost sharing).

### Costs studied among specific populations

Different OOP costs were studied among specific subpopulations, but insurance cost sharing for medication was still the most widely studied cost across many groups. None of the 5 articles limited to men studied child/dependent care costs, absenteeism, or transportation/travel, whereas 12, 20, and 22 of the 88 articles that were limited to women did.

For articles limited to populations with commercial insurance, the largest proportion studied insurance cost sharing for either nonmedication (54%) or medication (48%). For articles limited to either populations with Medicare or populations with Medicaid, the largest proportion studied insurance cost sharing for medication (55% and 46%, respectively). For the 14 articles limited to populations without insurance, the largest proportion studied absenteeism/lost wages (57%) and travel/transportation costs (57%).

Different OOP costs were also reported in different clinical settings. Articles that included inpatient care had the most variation in the type of OOP costs studied, with 28 distinct costs identified across 250 articles of which the largest proportion were transportation/travel costs (42%). In contrast, articles including telehealth had the least variation, with only 8 distinct types of costs identified across 20 articles that included at least 1 cost incurred in a telehealth setting.

The most frequently studied cost in articles that included telehealth was transportation (*n* = 16), followed by absenteeism/lost wages (*n* = 7). Although this may seem counterintuitive, some of these articles compared OOP costs in a telehealth setting with those in in-person clinical settings (eg, outpatient/ambulatory care). Thus, not all of the costs studied may have been incurred related to a telehealth encounter.

### Stratifying OOP costs

More than half of the articles (*n* = 460) stratified types of OOP costs by demographic, clinical, economic, and/or social characteristics. The most frequently applied stratification for OOP costs was insurance type (*n* = 184), with 16 unique OOP costs being stratified by insurance type. One-hundred and fifty articles stratified OOP costs by health condition, studying 25 different subcategories of OOP costs. In addition, 67 articles stratified OOP costs by race and 53 by ethnicity. For articles studying direct medical costs, 52% stratified their populations in some way compared with 47% of articles studying indirect costs and 29% studying direct nonmedical costs.

Articles also stratified other SDOH factors, including but not limited to income (*n* = 82), education level (*n* = 37), employment status (*n* = 19), history of incarceration (*n* = 1), nativity/immigration status (*n* = 2), access to IT (*n* = 1), and food insecurity (*n* = 4).

## Discussion

Available research examining OOP costs is often limited to insurance-related payments, which do not represent the full economic burden of accessing health care. Policies and interventions developed based on these definitions are, therefore, not able to address the totality of costs faced by patients and unpaid caregivers in the United States. The purpose of this systematic literature review was to create a comprehensive catalog of the real-world OOP health care expenditures experienced by patients and unpaid caregivers.

The findings add further nuance to current definitions of OOP costs, frame the concept of affordability from the patient perspective, and show the variety of approaches that researchers are taking to conceptualize and quantify OOP costs. Promulgating a more comprehensive understanding of OOP costs, including direct nonmedical and indirect costs, can help patients and families prepare for and navigate financial barriers to care. It can also guide health care providers, life science companies, policymakers, and other stakeholders in identifying opportunities to reduce financial burden and promote more accessible, equitable, and patient-centered care.

Almost all studies measuring OOP costs only capture a few components, thus systematically underestimating patient burden. The literature on OOP costs is also more developed in some areas than others. Insurance cost sharing is the most extensively studied cost type—more than half of the articles reviewed (*n* = 449) studied medication cost sharing, nonmedication cost sharing, and/or premiums. Similarly, oncology was the clinical area with the highest number of articles and most diversity in OOP costs, and inpatient was the most frequently explored clinical setting. But other topics are less well studied, and not all costs are explicitly defined as “out-of-pocket.”

Of the 817 articles included in this review, 42% did not classify the studied costs as “out-of-pocket,” suggesting significant variation in what is considered an OOP health care cost. Indirect and direct nonmedical costs were less likely to be referred to as “out-of-pocket” and are often missed when defining OOP costs. For example, funeral costs were only captured in 1 article, yet they represent a significant financial burden, especially among populations with limited financial resources.^37^ In contrast, direct medical costs, especially insurance-related costs, are more widely recognized as OOP.

This research shows that claims/billing data represented the second most common data source across included articles (*n* = 305). The significant representation of claims/billing data sources in articles indicates that individuals without insurance are being missed in many OOP cost estimates. These articles, by definition, also miss expenditures made outside of an insurance context, including many direct nonmedical and indirect costs such as child/dependent care costs and absenteeism/lost wages.

The authors also found a substantial difference in the number of articles studying OOP costs to unpaid caregivers (*n* = 184, 23%) compared with articles studying OOP costs to patients (*n* = 762, 93%). Given that the population of unpaid caregivers in the United States is predicted to rise exponentially in the coming years, it will be important for patients, caregivers, assistance/advocacy groups, and policymakers to recognize and address these costs across populations, settings, and therapeutic areas.^38^

A small fraction of articles limited their study population to historically excluded and medically underserved groups or stratified their results to expose disparities among these populations.

Although evidence shows that language barriers are associated with lower quality of care, longer hospital stays, and higher readmission rates, which may ultimately increase OOP costs to patients, only 25 articles specifically studied populations speaking non-English languages.^39^ Moreover, language interpreter services may increase costs for the health care system.^40^

While 67 articles did stratify their results by race, only 11 articles focused on a specific racial group. Lack of research on specific racial and ethnic groups is also a critical gap, given the disproportionate OOP burden among Black and Hispanic adults.^4^

Similarly, costs for transgender populations are not well studied. Insurance coverage for gender-affirming care varies by state, creating high OOP costs that prevent many transgender individuals from accessing the care they need.^41,42^ The recent proliferation of anti-transgender policies in many states may create additional burdens for transgender individuals and/or their parents, requiring them to travel out of state or seek care outside of insurance networks.^43^ Discrimination and marginalization may also impede future research if people do not feel that research participation is safe.^44,45^

Among articles limited by gender, substantially more articles focused on costs to women and had greater variation in the types of cost to women compared with men. Only articles focused on women studied direct nonmedical and indirect costs, including child/dependent care costs, which aligns with research that shows that women often face a significantly larger burden of household care duties.^46^ However, it could also indicate a bias by researchers, who may be more likely to study costs related to household and family care among women than among men.

### Impact on health care policy and recommendations

A comprehensive measure of OOP burden based on patients' and unpaid caregivers' real-world experiences can support stakeholders such as payers and policymakers in understanding what affordability means to patients and society. A multifaceted understanding of OOP health care costs can inform policy solutions to reduce barriers to care, especially among vulnerable and marginalized populations.

This is particularly important considering the recent March 2023 *Braidwood Management versus Becerra* ruling, which may eliminate an Affordable Care Act requirement for insurers and employers to cover certain preventive services without cost sharing and could lead to additional OOP costs or access challenges for patients.^9^

These findings emphasize the need for public and private policymakers to consider the full catalog of OOP costs when making coverage decisions to ensure that they are inclusive of all the different costs faced by patients and their families. Recent federal policies have paved the way for Medicare Advantage (MA) and prescription drug plans—outside of a demonstration—to tailor benefits for a subpopulation in an effort to improve access and mitigate medication and treatment OOPs for those beneficiaries.

For example, the Inflation Reduction Act of 2022 (IRA) capped annual OOPs for medications to $2000, down from $3100.^47^ The IRA also introduced monthly copay caps on insulin (predeductible) within Medicare Part D and removed cost sharing for vaccines covered under Part D.^48^ A July 2023 Centers for Medicare and Medicaid Services (CMS) publication reported that in 2025, these IRA Part D drug-related provisions will lead to an estimated $7.4 billion reduction in OOP spending for 18.7 million enrollees (36% of Part D beneficiaries) per year.^49^ This translates to about $400 per person among those enrollees who have OOP savings.

As another example, the 2019 MA and Part D Rate Announcement and Call Letter reinterpreted the statute to both expand the scope of health-related supplemental benefits and allow for plans to offer targeted cost sharing, benefits, and services as long as “similarly situated individuals are treated uniformly.”^50^ However, this review suggests that although these policies indicate progress, they are missing an opportunity to address nonmedical and indirect costs, which could ensure a greater positive impact on health by reducing disparities in access to care.

Regulators (eg, CMS, the Center for Medicare and Medicaid Innovation [CMMI]) also have a role in creating incentive structures, such as quality reporting programs and value-based payment programs, that reward health care practitioners and payers for providing or covering a wider array of services or reducing OOP costs. The CMMI Value-Based Insurance Design (VBID) model, for example, encourages MA plans to structure benefit design so that OOP costs are lower for “higher-value” activities.^51^ Under the VBID model, MA plans can also provide patients with tailored supplemental benefits such as transportation and grocery assistance.^52^

CMMI models test new ways of paying for health care that can catalyze broader policy change. For example, the success of the Part D Senior Savings Model led to the Part D and Part B copay caps on insulin and may become a template for other conditions.^53^

Regulators outside of HHS can also impact OOP. The US Treasury Department, for example, paved the way for expanding predeductible preventive care by issuing IRS Notice 2019–45, which provides a path to access Health Savings Accounts funds to cover the OOP costs associated with designated preventive care for those with high-deductible health plans (HDHPs) and chronic conditions such as asthma, diabetes, and hypertension.^54^ Considering about 56% of Americans were enrolled in HDHPs in 2021,^55^ such access could help millions of individuals receive care while managing costs. A 2021 survey of 354 large employers reported that 76% added predeductible coverage as a result of IRS Notice 2019-45, enhancing access to chronic disease services for millions of Americans.^56^

Direct nonmedical and indirect OOP costs not only impact patients and families, but also society overall. For example, lost income from missing work to seek health care affects the patient directly, whereas loss of productivity also impacts their employer. An analysis by the Integrated Benefits Institute found that chronic illnesses and injuries among the US workforce costs employers $575 billion a year due to absences and lost productivity.^57^

A 2023 Commonwealth Fund Survey found that medical debt incurred from OOP costs undermines patients' ability to pay for other expenses.^58^ Medical debt and income lost to illness has also been linked to >60% of bankruptcies in the United States.^59^ It follows that these costs may thus contribute to individual needs for taxpayer-funded government assistance for affordable food, housing, and health care.

Both payers and employers should consider exploring models that cover direct nonmedical and indirect expenses or population health models that offer the flexibility for providers to support patients and caregivers with services designed to defray the range of OOP costs. Employers can further utilize these findings to augment current practices and introduce new policies to support productivity and health outcomes.

For example, only 11% of workers in the United States report having employer-provided childcare, and those with lower incomes were less likely to receive that benefit.^60^ Integrating benefits related to childcare, eldercare, or care for adult children with disabilities (including respite care) on a broader scale may also help reduce OOP costs and disparities.

At the state level, some Medicaid Managed Care Organizations have begun incorporating coverage for nonmedical costs, including transportation for health services, linking members to food and housing, and supporting employment and educational services.^61–63^ Studies show addressing these SDOH factors can improve patient outcomes and lower costs to individuals and health systems.^64^ Expanding and standardizing these coverage policies can further help reduce inequities and burdens faced by marginalized populations.

The findings related to the variety of caregiver OOP costs suggest the need for accessible programs that support caregivers. For example, some states allow a family member or friend to become a paid caregiver if the person they are caring for already receives Medicaid.^65^ However, each state has different requirements, making it difficult to navigate and access these benefits.

Creating federal standards that support Medicaid-funded caregiver programs can help mitigate administrative barriers to accessing support and reduce financial burden on unpaid caregivers. Programs that employ community health workers can also help reduce caregiver burden, especially within marginalized communities, by offering low-cost resources and navigation assistance programs for caregivers.

In the US health care ecosystem, many types of organizations play a critical role in how patients access and afford medications, a critical facet of care. Some of these include payers, medical supply and pharmaceutical distributors, and life sciences companies. Many organizations already provide opportunities to alleviate some of this burden—including patient assistance programs or drug donation initiatives for those most at risk for high costs. Novel payment mechanisms, such as value-based contracting, are another opportunity for exploration. Because the challenges are multifaceted and the ecosystem of payment and delivery is evolving, the definitions of OOP costs and the solutions these organizations deploy to address them must also evolve.

### Limitations

This review was limited to care settings in the United States. Although the findings from this review cannot be generalized beyond the United States because individuals may experience different types of expenses in different countries, the identified OOP costs may be relevant to stakeholders in international settings.

Moreover, the data extraction was restricted by the methods and language used by the authors of each study and cannot account for variations in how individual studies defined, combined, and studied OOP costs. For example, if an article captured parking-related costs within transportation/travel costs and did not explicitly discuss parking-related expenditures, the OOP costs would be captured as transportation/travel cost.

In addition, the current review only identified 20 articles that studied OOP costs associated with telehealth. The authors did not explore how telehealth may have transformed OOP costs for patients and caregivers. The authors also included articles through March 2022; hence, any telehealth-related articles published afterward may not have been captured. Given the rapid expansion of telehealth during the COVID-19 pandemic, research should explore if and how telehealth OOP costs, such as technology-related costs, impact patient and unpaid caregiver perspectives, decisions, and health outcomes.^66^

Similarly, due to the review timeframe, no included articles discuss OOP repercussions of overturning Roe versus Wade, which eliminated constitutional protection for abortion rights. For instance, pregnant people may face increased financial burden such as expenses for travel, accommodation, and childcare if they must travel out of state to receive abortion care.^67^

Although a strength of this review is its consideration of OOP across a heterogeneous selection of research populations, the review, by definition, is restricted to examining existing literature. Hence, the limitations in current literature also carry through into the findings.

For example, limited articles included in the review used qualitative data. Although quantitative research is important to assess the magnitude and impact of costs and SDOH barriers, qualitative research is key in understanding how patients and unpaid caregivers experience OOP costs and consider trade-offs in their decision-making processes. Qualitative research can further help illuminate additional OOP expenses, expose unique experiences of marginalized and minority groups, and inform interventions that are more relevant to patients and unpaid caregivers.

In addition, to the extent that historically marginalized or underserved populations are underrepresented in research, these populations (eg, uninsured people, unhoused people, gender and sexual minorities) are also not well represented in this study.^68^

### Directions for future research

Given the disproportionate amount of literature on direct medical costs, there is a need for more research into direct nonmedical and indirect costs across therapeutic areas and settings, and how these costs impact patient decisions and behaviors. This review did not analyze the impact of OOP costs on patient decisions or behaviors, which is an important aspect in understanding the full impact that OOP costs have on patients and caregivers. Literature shows that lowering OOP costs, including cost sharing, improves medication adherence and utilization of routine treatment such as contraceptives.^13,69^

Because much of the literature to date focuses on insurance-related costs, future research should investigate the impact of direct nonmedical and indirect costs on patient decisions. Additional research should also focus on patient and provider education and techniques for ensuring that patients and unpaid caregivers are informed not only about the direct medical costs but also the direct nonmedical costs and indirect costs when seeking care. This information can also assist in shared decision making, especially in clinical areas where multiple treatment pathways are available.^70^

Considering the variety of approaches to measuring OOP costs, researchers may benefit from a standardized OOP measurement framework and/or assessment tool that encompasses all OOP costs experienced by both patients and unpaid caregivers to better compare and analyze costs across studies and populations. The catalog of OOP costs presents a useful starting point to support future frameworks and tools.

Researchers should also use a broader OOP cost definition, including elements found in the catalog provided in this review, in future studies of patient and unpaid caregiver costs. Lastly, costs are only 1 driver of health behaviors and outcomes. Although lowering costs is an effective way to improve access and drive health behaviors, researchers and interventions should also consider how patient and/or caregiver perception of affordability impacts health care decisions.

## Conclusions

OOP costs are multifactorial, inconsistently measured or undermeasured, and underestimated. Even when OOP costs are consistently measured, there is variation in how they are operationalized, collected, and reported. OOP costs not only impact individual patients, families, and unpaid caregivers, but also economic growth and the society in general. In addition, patient decisions to delay or omit needed care due to costs may result in much greater future costs for emergency care, increasing the burden on private and government payers.

As OOP health care spending continues to increase and policymakers consider efforts to minimize financial barriers to care, it is imperative that the full scope of OOP costs faced by patients and unpaid caregivers is documented and understood to support effective interventions to reduce financial burden and increase access to needed care, especially among historically marginalized groups that face disproportionately higher OOP costs and additional challenges to accessing health care.

## Supplementary Material

Supplemental data

Supplemental data

Supplemental data

## Supplementary Material

Supplementary Appendix SA1

Supplementary Appendix SA2

Supplementary Appendix SA3

## Notes

^*^
$24 billion in 1970 translates to about $166 billion when adjusted for inflation to 2021 currency.
