# Supplementary material for: Expanding the Catalog of Patient and Caregiver Out-of-Pocket Costs: A Systematic Literature Review
Source: Popul Health Manag. 2024 Feb 6;27(1):70–83. doi: 10.1089/pop.2023.0238 (PMC10877382; doi:10.1089/pop.2023.0238)
Supplement: Supplemental data [file Suppl_AppendixSA1.docx]

# Expanding the Catalog of Out-of-Pocket Costs Supplemental Appendix 1: Search Strings

**Appendix 1A: PubMed Search String**

| **Populations & Intervention** | ((("Patient*" [tiab] OR "caregiver*" [tiab] OR "famil*" [tiab] OR “beneficiar*” [tiab] OR “household” [tiab] OR “enrollee*” [tiab]) AND (((“Transportation” [tiab] OR “parking” [tiab] OR “food” [MeSH] OR “accommodation” [tiab] OR “child care” [MeSH] OR “Nonprescription Drugs” [MeSH]) AND (“cost*” [tiab] OR “expens*” [tiab] OR “expend*” [tiab] OR “spend*” [tiab])) OR “absenteeism” [MeSH] OR “presenteeism” [MeSH] OR “lost wages” [tiab] OR “indirect cost*” [tiab])) OR “Out-of-Pocket” [tiab] OR “OOP” [tiab]) |
| --- | --- |
| **Limitations/ Exclusions** | NOT (((Case Reports [pt]) OR (Biography [pt]) OR (Video-Audio Media [pt]) OR (Letter [pt]) OR (Practice Guideline [pt]) OR (Clinical Trial Protocol [pt]) OR (Clinical Trial, Phase I [pt]) OR (Clinical Trial, Phase II [pt]) OR (Clinical Trial, Phase III [pt]) OR (Clinical Trial, Phase IV [pt]) OR (Comment [pt]) OR (Editorial [pt])) OR ("Animals"[Mesh] NOT "Humans"[Mesh]) OR (“Plants”[MeSH]) OR ("Aquaculture"[MeSH]) OR (“Candy” [MeSH] OR “Chocolate” [MeSH] OR “Condiments” [MeSH] OR “Crops, Agricultural” [MeSH] OR “Edible Insects” [MeSH])) |
| **Filters** | AND ("2017/04/01"[Pdat] : "2022/03/31"[Pdat]) AND (english[Filter]) AND (fft[Filter]) |

**Appendix 1B: Embase Search String**

| **Populations & Intervention** | (('patient*':ti,ab OR 'caregiver*':ti,ab OR 'famil*':ti,ab OR 'beneficiar*':ti,ab OR 'household*':ti,ab OR 'enrollee*':ti,ab) AND (('transportation':ti,ab OR 'parking':ti,ab OR 'food'/de OR 'accommodation':ti,ab OR 'child care'/de OR 'nonprescription drugs'/de) AND ('cost*':ti,ab OR 'expens*':ti,ab OR 'expend*':ti,ab OR 'spend*':ti,ab) OR 'absenteeism'/de OR 'presenteeism'/de OR 'lost wages':ti,ab OR 'indirect cost*':ti,ab) OR 'out-of-pocket':ti,ab OR 'oop':ti,ab) |
| --- | --- |
| **Limitations/ Exclusions** | NOT ('letter'/it OR ‘editorial’/it OR ([animals]/lim NOT [humans]/lim) OR 'animal cell'/de OR 'animal experiment'/de OR 'animal model'/de OR 'animal tissue'/de OR 'case report'/de OR 'human cell'/de OR 'human tissue'/de OR 'in vitro study'/de OR 'in vivo study'/de OR 'nonhuman'/de OR 'clinical trial protocol'/de OR ‘phase 4 clinical trial’/de OR ‘phase 3 clinical trial’/de OR ‘phase 2 clinical trial’/de OR ‘phase 1 clinical trial’/de OR 'practice guideline'/de OR 'candy'/de OR 'chocolate'/de OR 'condiments'/de OR 'crop'/de OR 'edible insect'/de) |
| **Filters** | [english]/lim AND [abstracts]/lim NOT 'conference abstract'/it AND [01-04-2017]/sd NOT [01-04-2022]/sd NOT [clinical trial number]/lim |

**Appendix 1C: Cochrane Central Register of Controlled Trials (CENTRAL) Search String**

| **Populations & Intervention** | ((("patient" OR "caregiver" OR "family" OR "beneficiary" OR "household" OR "enrollee"):ti,ab,kw AND (((("transportation"):ti,ab,kw OR ("parking"):ti,ab,kw OR (MeSH descriptor: [Food] explode all trees) OR ("accommodation" OR “accommodations”):ti,ab,kw OR (MeSH descriptor: [Child Care] explode all trees) OR (MeSH descriptor: [Nonprescription Drugs] explode all trees)) AND ("cost" OR "expense" OR "expenditure" OR "spend"):ti,ab,kw) OR ((MeSH descriptor: [Absenteeism] explode all trees) OR (MeSH descriptor: [Presenteeism] explode all trees) OR ("lost wages"):ti,ab,kw OR ("indirect cost" OR "indirect costs"):ti,ab,kw))) OR ("OOP" OR "out-of-pocket"):ti,ab,kw |
| --- | --- |
| **Limitations/ Exclusions** | NOT ((MeSH descriptor: [Animals] explode all trees) NOT (MeSH descriptor: [Humans] explode all trees)) AND (MeSH descriptor: [Plants] explode all trees) AND (MeSH descriptor: [Aquaculture] explode all trees) AND (MeSH descriptor: [Candy] explode all trees) AND (MeSH descriptor: [Chocolate] explode all trees) AND (MeSH descriptor: [Condiments] explode all trees) AND (MeSH descriptor: [Crops, Agricultural] explode all trees) AND (MeSH descriptor: [Edible Insects] explode all trees) |
| **Filters** | with Cochrane Library publication date between Apr 2017 and Mar 2022 |
