# Supplementary material for: Expanding the Catalog of Patient and Caregiver Out-of-Pocket Costs: A Systematic Literature Review
Source: Popul Health Manag. 2024 Feb 6;27(1):70–83. doi: 10.1089/pop.2023.0238 (PMC10877382; doi:10.1089/pop.2023.0238)
Supplement: Supplemental data [file Suppl_AppendixSA2.docx]

# Expanding the Catalog of Out-of-Pocket Costs Supplemental Appendix 2

**Appendix 2: SPIDER Framework**

| **Sample** | United States patients and/or unpaid caregivers |
| --- | --- |
| **Phenomenon of Interest** | Real-world experiences interacting with the health care system  Secondary variables for extraction and synthesis (not criteria for inclusion/exclusion) - Patient/caregiver characteristics studied in relation to out-of-pocket (OOP) costs. Potential characteristics may include demographic (eg, race), clinical (eg, health condition), social (eg, marital status), and economic (eg, income) factors |
| **Design** | - Systematic reviews (or systematic scoping reviews) with and without meta-analysis - Randomized controlled trials - Non-randomized controlled trials (eg, prospective cohort studies) - Observational studies with controls (eg, retrospective studies, case-control studies) - Observational studies without controls (eg, cohort studies without controls, case series) - Instrument validation studies |
| **Evaluation** | Defined OOP costs (direct and/or indirect) associated with real-world health care experiences specific to actual US patients and/or unpaid caregivers. Examples include:   - Total OOP costs - Copayments/co-insurance/deductibles - Work-related absenteeism/lost wages in relation to treatment - Accommodations - Caregiver expenses - Non-covered/out-of-network costs - Parking-related costs - Transportation/travel - Meals - Over the counter products - Childcare |
| **Research type** | Qualitative |
| **Other** | - Exclude literature reviews - Exclude conference abstracts - Exclude study protocols - Exclude clinical trials (ie, article categorized as a “clinical trial” by the author) - Exclude cost-effectiveness studies - Exclude commentaries, perspective pieces, opinion articles, and narrative reviews with no original research - Exclude hypothetical results (eg, discrete choice experiments, willingness to pay studies) - Exclude policy analysis that predicts future impact of policies - Exclude articles examining theoretical frameworks for drug pricing - Exclude articles assessing drug pricing variation (eg, by geography) - Exclude systematic literature reviews that focus on only articles that would otherwise be excluded - Exclude non-human articles - Exclude non-English language publications |
