# Supplementary material for: Expanding the Catalog of Patient and Caregiver Out-of-Pocket Costs: A Systematic Literature Review
Source: Popul Health Manag. 2024 Feb 6;27(1):70–83. doi: 10.1089/pop.2023.0238 (PMC10877382; doi:10.1089/pop.2023.0238)
Supplement: Supplemental data [file Suppl_AppendixSA3.docx]

# Expanding the Catalog of Out-of-Pocket Costs Supplemental Appendix 3

**Appendix** **3: Summary of Article Characteristics**

| **Article Characteristics** | **Number of Articles** |
| --- | --- |
| **Was Article Objective to Study Out-of-Pocket (OOP) Costs?** | |
| No | 141 |
| Yes | 676 |
| **Study Type** | |
| Case series/case study | 4 |
| Case-control study | 5 |
| Cross-sectional study | 167 |
| Instrument validation studies | 3 |
| Mixed methods study | 25 |
| Prospective and/or retrospective cohort study | 272 |
| Qualitative research, other (including qualitative observational or descriptive research) | 73 |
| Quasi-experimental study | 15 |
| Randomized controlled trial | 3 |
| Systematic review (with/without meta-analysis) | 56 |
| Other | 13 |
| *181 articles did not specify a study type.* | |
| **Data Source*** | |
| Claims/billing data | 305 |
| Demographic data | 11 |
| Electronic health record | 63 |
| Focus groups/interviews | 107 |
| Other articles/studies | 58 |
| Other clinical or pharmacy data | 69 |
| Social media listening | 2 |
| Surveys | 337 |
| *12 articles used a data source other than those specified here.* | |
| **Research Participants for Surveys, Focus/Groups and Interviews, or Other Qualitative Research*** | |
| Administrators | 6 |
| Clinicians | 50 |
| Family/unpaid caregiver | 100 |
| Other health care staff | 4 |
| Patients | 355 |
| *4 articles included participants other than those specified here.* | |
| **State*** | |
| All | 261 |
| Alaska | 7 |
| Alabama | 9 |
| Arkansas | 6 |
| Arizona | 13 |
| California | 44 |
| Colorado | 19 |
| Connecticut | 11 |
| Delaware | 7 |
| Florida | 18 |
| Georgia | 17 |
| Hawaii | 4 |
| Iowa | 9 |
| Idaho | 6 |
| Illinois | 21 |
| Indiana | 7 |
| Kansas | 9 |
| Kentucky | 11 |
| Louisiana | 9 |
| Massachusetts | 30 |
| Maryland | 12 |
| Maine | 9 |
| Michigan | 21 |
| Minnesota | 12 |
| Missouri | 10 |
| Mississippi | 9 |
| Montana | 5 |
| North Carolina | 21 |
| North Dakota | 5 |
| Nebraska | 7 |
| New Hampshire | 9 |
| New Jersey | 16 |
| New Mexico | 9 |
| Nevada | 7 |
| New York | 26 |
| Ohio | 18 |
| Oklahoma | 5 |
| Oregon | 15 |
| Pennsylvania | 24 |
| Rhode Island | 12 |
| South Carolina | 11 |
| South Dakota | 6 |
| Tennessee | 14 |
| Texas | 26 |
| Utah | 7 |
| Virginia | 13 |
| Vermont | 7 |
| Washington | 24 |
| Wisconsin | 15 |
| West Virginia | 5 |
| Wyoming | 4 |
| *336 articles did not specify a state.* | |
| **Region*** | |
| All | 428 |
| Midwest | 48 |
| Northeast | 61 |
| South | 71 |
| West | 66 |
| *175 articles did not specify a state or region.* | |
| **Limited by Age** | |
| Adult (18 years or older) | 550 |
| Pediatric (<18 years) | 88 |
| Both but not all age groups | 45 |
| **Limited by Language** | |
| English | 94 |
| English; Spanish | 22 |
| English; French | 1 |
| English; Spanish; Vietnamese | 1 |
| Hmong | 1 |
| **Limited by Race/Ethnicity** | |
| American Indian or Alaska Native | 1 |
| Asian | 1 |
| Asian; White | 1 |
| Black or African American | 2 |
| Black or African American; Hispanic or Latino | 1 |
| Black or African American; White | 3 |
| Hispanic or Latino | 2 |
| **Limited by Gender** | |
| Men | 5 |
| Women | 88 |
| Transgender/Nonbinary | 2 |
| **Limited by Insurance Status/Type** | |
| Commercial only | 157 |
| Medicaid only | 13 |
| Medicare only | 62 |
| No insurance only | 14 |
| **Therapeutic Area*** | |
| Behavioral and Mental Health | 65 |
| Cardiology | 44 |
| Dentistry | 8 |
| Dermatology | 11 |
| Gastroenterology | 22 |
| Hematology | 5 |
| Hepatology | 5 |
| Infectious disease/vaccines/immunology | 44 |
| Metabolism and Endocrinology | 45 |
| Nephrology | 11 |
| Neurology | 60 |
| Oncology | 177 |
| Ophthalmology | 11 |
| Orthodontics | 1 |
| Orthopedics | 32 |
| Otolaryngology | 9 |
| Primary care | 5 |
| Rare disease | 10 |
| Respiratory | 18 |
| Rheumatology | 19 |
| Urology | 66 |
| Women's health/Obstetrics & Gynecology | 49 |
| *172 articles did not specify a therapeutic area or were broadly applicable across areas.* | |
| **Setting*** | |
| Community center | 8 |
| Emergency department | 104 |
| Intensive care unit | 6 |
| Inpatient | 250 |
| Long-term care facility | 22 |
| Primary care/family medicine/general medicine/ provider office | 76 |
| Outpatient/ambulatory | 206 |
| Pharmacy | 187 |
| Specialty clinic | 106 |
| Telehealth | 20 |
| *55 articles included a setting other than those specified here.* | |

*These characteristics were not mutually exclusive, as articles could satisfy more than one field, and thus their sum exceeds 817.
